# Metabolomics Study of Guizhi Fuling Capsules in Rats With Cold Coagulation Dysmenorrhea

**DOI:** 10.3389/fphar.2021.764904

**Published:** 2021-10-28

**Authors:** Yu Zhang, Na Su, Weiyi Liu, Qingqing Wang, Jianguo Sun, Ying Peng

**Affiliations:** Key Lab of Drug Metabolism and Pharmacokinetics, State Key Laboratory of Natural Medicines, China Pharmaceutical University, Nanjing, China

**Keywords:** cold coagulation dysmenorrhea, guizhi fuling capsules, metabolomics, traditional Chinese medicine, arachidonic acid

## Abstract

Dysmenorrhea refers to a kind of uterine cramping pain that occurs in women during the period of menstrual. Guizhi Fuling Capsules are mainly used for the treatment of various pain syndromes and especially effective in treating primary dysmenorrhea. However, the research on its modern pharmacology and mechanism of action have not been thoroughly carried out. It is not clear about the main active ingredients, potential targets and metabolic pathways involved in its efficacy. Therefore, this research project employed estradiol benzoate sensitization combined with oxytocin pain to construct the cold coagulation syndrome dysmenorrhea model, observed the anti-dysmenorrhea effect of Guizhi Fuling Capsules, and used the metabolomics to explore its mechanism. The results showed that Guizhi Fuling Capsules could considerably reduce the number of writhing times in dysmenorrhea rats, increasing the level of PGE2 and β-EP and reducing the contents of PGF2α in rat serum. Pathological sections of uterus and ovaries also showed that Guizhi Fuling Capsules could significantly relieve endometrial hyperplasia and improve ovarian function. The LC/MS-based metabolomics of rat uterine indicated that the model group has a great deviation from the control group. Compared with the model group, the Guizhi Fuling Capsules group had a tendency to shift to the control group, and the main metabolic changes was mainly concentrated on saturated and unsaturated fatty acids. Among them, arachidonic acid is in a pivotal position, and the expression of its rate-limiting enzyme (COX-2) involved in its cyclooxygenase metabolic pathway was significantly up-regulated in the model group, but significantly decreased after the intervention of Guizhi Fuling Capsules. In conclusion, Guizhi Fuling Capsules can effectively relieve primary dysmenorrhea, and this effect may be attributed to the regulation effects of Guizhi Fuling Capsules on endogenous metabolism, such as inhibiting arachidonic acid converted to prostaglandins through downregulate the expression of COX-2, which plays an anti-inflammatory effect.

## Introduction

Dysmenorrhea refers to a kind of uterine cramping pain that occurs in women before or after the menstrual period (Shirvani et al., 2015). In recent years, with the increasement of social pressure, the incidence rate of dysmenorrhea is gradually growing. According to epidemiological statistics, about 50% of women suffered from dysmenorrhea, 10% of which had endured more severe menstrual cramps. This kind of pain not only affects individual’s quality of life, but also lower the level of happiness (Lefebvre et al., 2005). The treatment of dysmenorrhea in western medicine is mainly the application of non-steroidal anti-inflammatory drugs, which could merely provide temporary pain relief instead of fundamental treatment and additionally cause drug resistance, gastrointestinal adverse as well as liver and kidney damage. On the contrast, traditional Chinese medication focus on curing dysmenorrhea itself, where patients are diagnosed and treated based on the overall analysis of the disease and physical condition (Xiu-Hong et al., 2015).

Generally, dysmenorrhea could be categorized as primary dysmenorrhea and secondary dysmenorrhea, in which the primary one accounts for more than 90% (Meirong et al., 2020). Caused by excessive pathological uterine contraction, primary dysmenorrhea mainly occurs in women during their teenage years without any organic pelvic lesions, which pathogenesis is not clear. Syndrome of cold coagulation and blood stasis is one of the most common clinical classification of it. There are a variety of methods to establish the cold coagulation syndrome dysmenorrhea model: whole body freezing method (Shujing et al., 2018; Shujing et al., 2020), local frostbite method (Wang and Feng, 2000), wind cold environment method (Tang et al., 2000), ice water immersion method (Cheng et al., 2005), ice water immersion joint drug method (Yan et al., 2014; Xihong et al., 2017; Meng, 2018) and surgery plus drug method (Lu et al., 2010). However, the ice-water immersion method uses short-term overstimulation to build the model, and the local frostbite method ignores the overall concept, which both cannot fit well with the disease. Therefore, the cold coagulation dysmenorrhea model used in this experiment is established on the basis of the traditional dysmenorrhea model using the method of whole body freezing. This model comes from the dysmenorrhea animal model preparation specifications issued by the Chinese Society of Traditional Chinese Medicine and the Professional Committee of Experimental Pharmacology of Traditional Chinese Medicine in 2018 (medicine and Medicine., 2018). It conforms to the modeling ideas and characteristics of the TCM syndrome model and has been adopted by a large number of researchers.

Guizhi Fuling Capsules evolved from Guizhi Fuling Pills in “The Synopsis of the Golden Chamber” by Zhang Zhongjing in the Eastern Han Dynasty (Yunxi et al., 2016). It is mainly composed of Chinese medicinal materials such as Guizhi, Poria, White Peony, Peony Bark, and Peach Kernel. Jiangsu Kangyuan Pharmaceutical Co., Ltd. (Nanjing, Jiangsu, China) has continuously improves the production process of Guizhi Fuling Capsules, and now its production of Guizhi Fuling Capsules has successfully overcomes the unstable characteristics of traditional Chinese medicine preparations (Qingli et al., 2019). Guizhi Fuling Capsules have a variety of pharmacological effects such as anti-inflammatory, analgesic, anti-tumor, smooth muscle regulation, endocrine regulation and immunity enhancement. It is mainly used for gynecological indications, such as the treatment of dysmenorrhea, uterine fibroids, dysfunctional uterine bleeding, menorrhagia, amenorrhea, endometriosis, and some symptoms related to ovarian cysts (Yan et al., 2014). Meanwhile, studies have shown that the extract of Guizhi Fuling Capsules can inhibit the activity of protein tyrosine kinases and exert anti-tumor effects (Lianhua et al., 2012). Guizhi Fuling Capsules has definite curative effect, less side effects and adverse reactions, and is the preferred drug for the treatment of gynecological related diseases. However, the main active ingredients of Guizhi Fuling Capsules for anti-dysmenorrhea, the metabolic pathways involved and the target of the drug effect are not clear. Due to the complexity of the traditional Chinese medicine compound, it has not carried out in-depth modern pharmacology and the mechanism of action.

The objects of metabolomics research are small molecular compounds in biological samples, systems, tissues or cells in the body. Metabolomics is an emerging discipline that uses modern analytical methods such as chromatography and mass spectrometry to determine qualitative/quantitative information of endogenous compounds. The changes from these endogenous substances can reflect the interaction between the inside and outside of the organ or system, which is to reflect the physiological changes in a certain pathological process as a whole. In recent years, with the advancement of analytical methods such as HPLC and GC-MS, metabolomics research has developed rapidly, especially in the interpretation of traditional Chinese medicine prescriptions, pharmacological effects of traditional Chinese medicine and pharmacological effects of active ingredients in natural medicine. Metabolomics is also an important method on the road of Chinese medicine going abroad and being recognized and accepted by the world. Metabolomics can reflect the changes of small molecule metabolites in the body at an overall level. Therefore, this research will explore the possible mechanism of Guizhi Fuling Capsules’s anti-dysmenorrhea effect by using metabolomics technology.

## Methods

### Materials and Reagents

Estradiol benzoate was purchased from Aladdin. Oxytocin injection and soybean oil was purchased from Maclean. Guizhi Fuling Capsules are provided by Kangyuan Pharmaceutical. 5-13C-glutamine (Cambridge Isotope Laboratories, Andover, MA, United States); PGF2α kit, PGE2 kit and β-EP kit were purchased from Nanjing Jiancheng Bioengineering Research Institute.

### Experimental Animals

Three-months-old female SD rats (SPF grade) were purchased from Shanghai SIPPR-BK Laboratory Animal Co., Ltd. (Shanghai, China). The rats were fed with standard food with 12/12 h light/dark cycle and drink freely. Animal experiments were approved by the Ethics Committee of China Pharmaceutical University. We did our best to reduce animal suffering and reduce the number of animals used.

SD rats were divided into control group, model group, Guizhi Fuling administration group, six in each group. Except for the blank group, in the 10-day modeling cycle, the others were injected subcutaneously with estradiol benzoate at a dose of 2.5 mg/kg on the first and last days and at a dose of 1 mg/kg from day 2 to day 9. After disassembling the capsule shell of the Guizhi Fuling Capsule, grind the content and suspend it with 0.5% CMC-Na to prepare a suspension with a concentration of 1 g/kg, which is equivalent to 10 times the human dosage. Guizhi Fuling Capsules were administered orally for 10 days, and the time of each administration was half an hour after the injection of estradiol benzoate. After the administration, the rats were placed in an ultra-low temperature refrigerator (−20°C) for 2 h, and it was opened for ventilation for 5 s at 1 h. On the 10th day, 1 h after subcutaneous injection of estradiol benzoate, oxytocin was injected intraperitoneally at a dose of 2 U/kg. Observe the number of twists in the next 30 min.

### Sample Preparation

After successful modeling, the rats were bled from the femoral vein and sacrificed. Serum was collected for kit detection, while the uterine tissue was collected for related determinations. Accurately weigh and pulverize 30 mg of uterine tissue, add 900 μl of precipitant (15 μg/ml C13-glutamine in 80% methanol water) and two zirconium beads to prepare a homogenate. The mixture was centrifuged at 20,000 g at 4°C for 10 min. Transfer 200 μl of supernatant to a 1.5 ml EP tube, evaporate to dryness, reconstitute the residue with 100 μl of 80% methanol water, centrifuge at 18,000 rpm at 4°C for 5 min, transfer 80 μl of supernatant to a sample bottle. Then inject 10 μl for HPLC-QTOF/MS analysis.

### HPLC-Q/TOF-MS-Based Metabolomics Assay

#### Chromatographic Conditions

Column: Waters XB ridge Amide 3.5 μm, 4.6 × 100 mm Column, column temperature: 40°C, water phase (A): 5 mM ammonium acetate ultrapure water, adjust pH to 9.0 (containing 5% acetonitrile) with ammonia, organic phase (B): Acetonitrile, flow rate: 0.4 ml/min, analysis time: 26.0 min, gradient elution: 0 ∼ 3.0 min (85% B), 3.0 ∼ 6.0 min (85 ∼ 30% B), 6.0 ∼ 15.0 min (30 ∼ 2% B), 15.0 ∼ 18.0 min (2% B), 18.0 ∼ 19.0 min (2 ∼ 85% B), 19.0 ∼ 26.0 min (85% B).

#### Mass Spectrometry Conditions

The HPLC system consisted of a LC-30A binary pump, a SIL-30AC autosampler and a CTO-30AC column oven (Shimadzu, Japan) coupled with a hybrid quadrupole time-of-fight tandem mass spectrometer (AB SCIEX Triple TOF 5600, Foster City, CA). Electrospray ionization (ESI) is used for MS detection in negative ion mode. The parameter settings are as follows: TOF MS scan, m/z: 50–1000Da; product ion scan, m/z 50–900 Da; Gas 1, 50 psi; Gas 2, 30 psi, CUR, 30 psi; TEM,500; ISVF, −4500 V; DP, −100 V; CE, −10 V; and CE in product ion scan (IDA), -35 V.

#### Data Acquisition and Data Analysis

The accurate mass was calibrated by the calibration delivery system (CDS), and automatic calibration was carried out every eight samples. Data exploration and peak area integration were performed with PeakView and MultiQuant 2.0 from AB SCIEX. All uterine tissue samples were mixed as quality control (QC) samples, and the QC samples were injected every ten samples to monitor the stability of the analysis. All detected compounds were identified by comparing the retention times and mass spectra (both the MS and MS/MS spectra) of the detected compound with a reference database established in our laboratory (Wang et al., 2018) and with other free online databases, such as MASSBANK (http://www.massbank.jp/index-e.html), METLIN (http://metlin.scripps.edu) and MS2T (http://prime.psc.riken.jp/lcms/ms2tview/ms2tview.htm). The raw data is the peak area of each compound, which is used to represent its content in the sample.

### Statistical Analysis

The metabolites detected by metabolomics analysis were relatively quantitative and needed to be normalized by internal standard to weight the peak area. In order to compare the differences of each group, the data was analyzed using SIMCA-P13.0 bit software, which mainly included data pre-processing, data correction, PCA analysis, PLS-DA analysis and screening of differential compounds. Thus, further processing of spectra, analysis of metabolic pathway, analysis of physiological and biochemical significance can be taken. Principal component analysis (PCA) is an unsupervised method used to illustrate the overall distribution of all samples. Partial least squares discriminant analysis (PLS-DA) is a supervised method used to confirm the general separation of groups. Orthogonal partial least squares discriminant analysis (OPLS-DA) is an extension of PLS-DA and is used to distinguish two groups and identify different metabolites. In addition, MetaboAnalyst (http://www.metaboanalyst.ca) was used for metabolomic pathway analysis based on differential metabolites.

The results were expressed as mean ± S.D. All data were analyzed using Graph Pad Prism software (Graph Pad, United States). Statistical levels were calculated using one-way analysis of variance (ANOVA). A *p*-value of less than 0.05 was considered significantly different.

## Results

### Evaluation of the Therapeutic Effect of Dysmenorrhea Model

#### Behavioral Indicators

**FIGURE 1:**
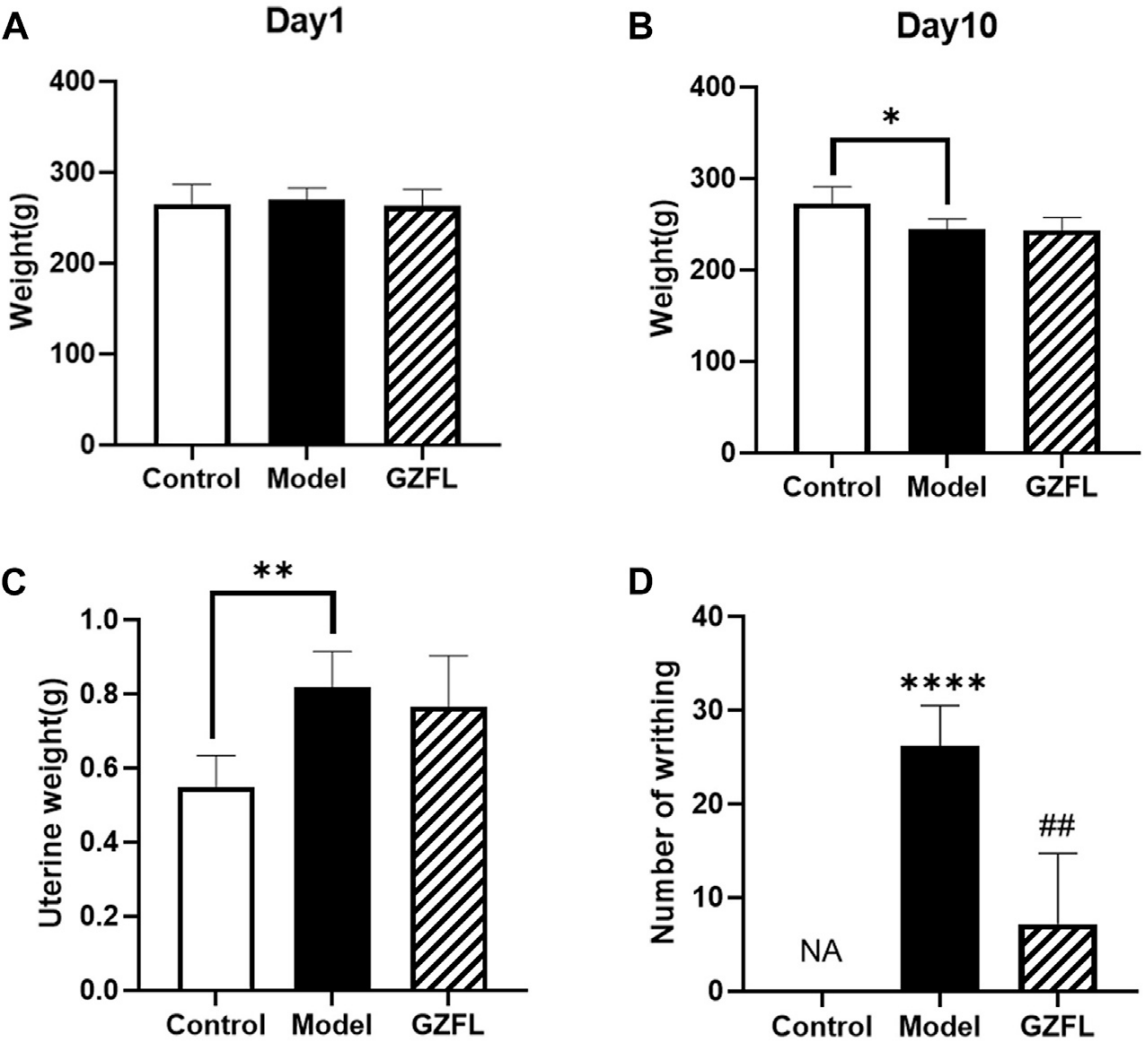
**(A)** The weight of the rat on day 1 of the experiment **(B)** The weight of the rat on day 10 **(C)** the weight of the uterus on day 10 of the experiment **(D)** the number of writhings recorded on day 10 of the experiment. ( $\bar{\text{x}}$ ±*s*, n = 6, compared with Control group: ^*^ *p* < 0.05 ^**^ *p* < 0.01 ^***^ *p* < 0.001 ^****^ *p* < 0.0001; compared with Model group: ^#^ *p* < 0.05 ^##^ *p* < 0.01 ^###^ *p* < 0.001 ^####^ *p* < 0.0001).

The Behavioral indicators is an important criterion for clinical diagnosis and judgment of female dysmenorrhea, it is an important technical indicator for the success of the design and preparation of dysmenorrhea models. The manifestations of writhing mainly include indentation on both sides of the abdomen, abdominal wall sticking down, buttocks raised, twisted body or hind limb extension. Each time the rat exhibits the above-mentioned writhing performance, it is considered that there is a writhing and recorded. In this experiment, rats in each group had basically the same body weight before administration while after 10 days of modeling, all rats in the experimental group except the control one lost weight **(** Figures 1A,B). Compared with the control group, the weight of the rat’s uterus increased after the administration of estradiol benzoate. (Figure 1C). The rats in the blank control group did not show any writhing symptoms, while that in the model group showed strong symptoms. As shown in Figure 1D, compared with the model group, the writhing frequency of the medical intervention groups was significantly reduced.

### Biochemical Indicators

**FIGURE 2:**
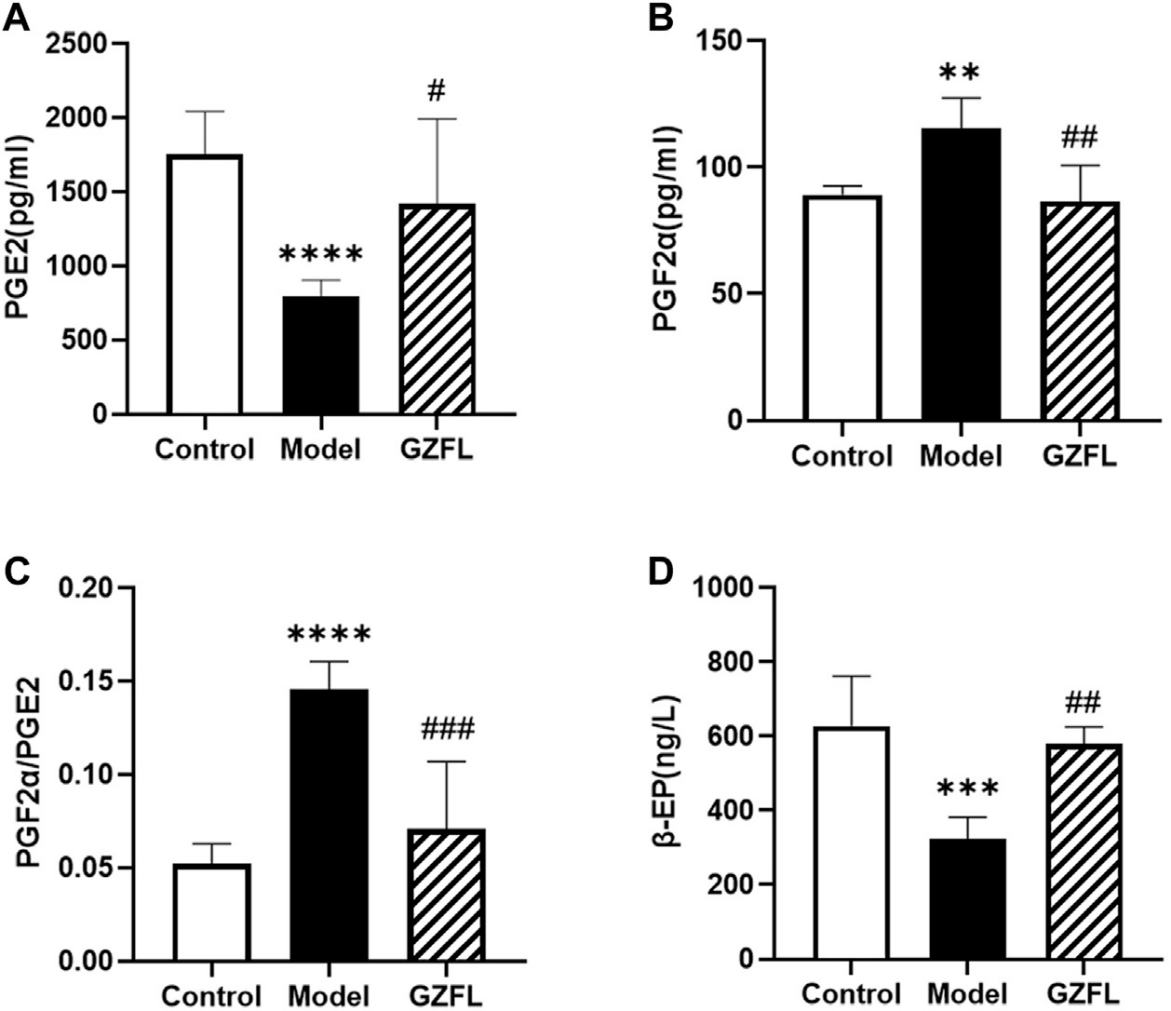
Serum biochemical indexes **(A)** PGE_2_ level **(B)** PGF_2α_ level **(C)** PGF_2α_/PGE_2_ ratio **(D)** β-EP level ( $\bar{\text{x}}$ ±*s*, n = 6, compared with control group: ^*^ *p* < 0.05 ^**^ *p* < 0.01 ^***^ *p* < 0.001 ^****^ *p* < 0.0001; compared with model group: ^#^ *p* < 0.05 ^##^ *p* < 0.01 ^###^ *p* < 0.001 ^####^ *p* < 0.0001).

As shown in Figure 2, the level of PGE2 in the serum of rats in the model group was significantly reduced, while that in the model group was significantly increased after administration. However, the level of PGF2α showed an opposite trend to that of PGE2. The content of PGE2α in the serum of rats in the model group was abundant, and it was significantly reduced after administration. Therefore, the ratio of PGF2α/PGE2 in the model group increased significantly, and decreased significantly after administration. The trends of β-EP and PGE2 are basically the same. Compared with the control group, the β-EP content of the model group reduced significantly, while that of the Guizhi Fuling group increased significantly.

### Pathological Indicators

**FIGURE 3:**
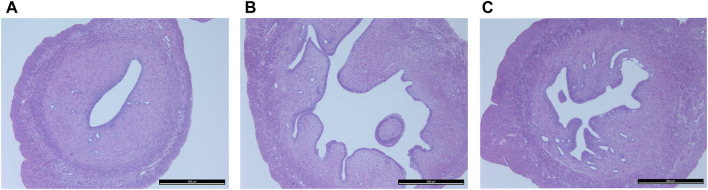
Histopathological section of uterus**(A)** Control group **(B)** Model group **(C)** GZFL group.

**FIGURE 4:**
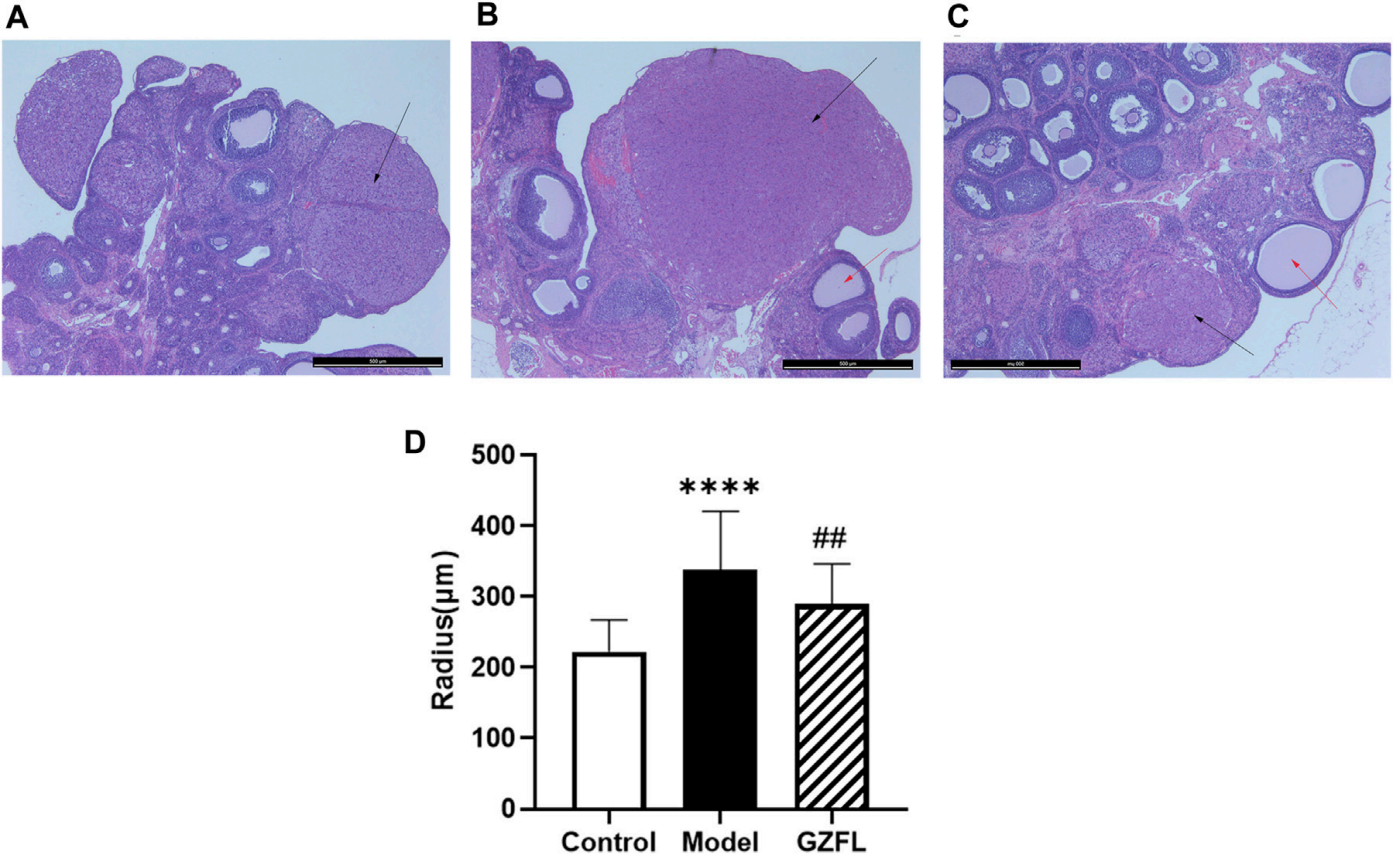
Typical ovarian histopathology section, The black arrow represents the corpus luteum, the red arrow represents atretic follicles **(A)** Control group **(B)** Model control group **(C)** GZFL group **(D)** Rat corpus luteum radius ( $\bar{\text{x}}$ ±*s*, n = 6, compared with control group: ^*^ *p* < 0.05 ^**^ *p* < 0.01 ^***^ *p* < 0.001 ^****^ *p* < 0.0001; compared with model group: ^#^ *p* < 0.05 ^##^ *p* < 0.01 ^###^ *p* < 0.001 ^####^ *p* < 0.0001).

Figure 3 shows histopathological sections of the uterus. In the control group, endometrial epithelial cells are complete, the thickness of endometrium and myometrium is normal, and uterine cavity is smooth. However, in the model group, the uterine structure is out of order, uterine cavity is not smooth and the endometrial hyperplasia is obvious. The uterine cavity of the Guizhi Fuling group tends to be smooth. Figure 4 shows typical pathological sections of the rat ovary. For ovarian tissue, the follicles of the control group are arranged in an orderly manner. While in the model group, the number of atresia follicles increases, the arrangement of follicles is disordered, after the treatment of Guizhi Fuling, the symptoms above are relieved. As shown in Figure 4D, compared with the control group, the corpus luteum radius of the model group increased by about 1.5 times, and the corpus luteum radius decreased by about 1.2 times after administration of Guizhi Fuling.

### Metabolomics Analysis

**FIGURE 5:**
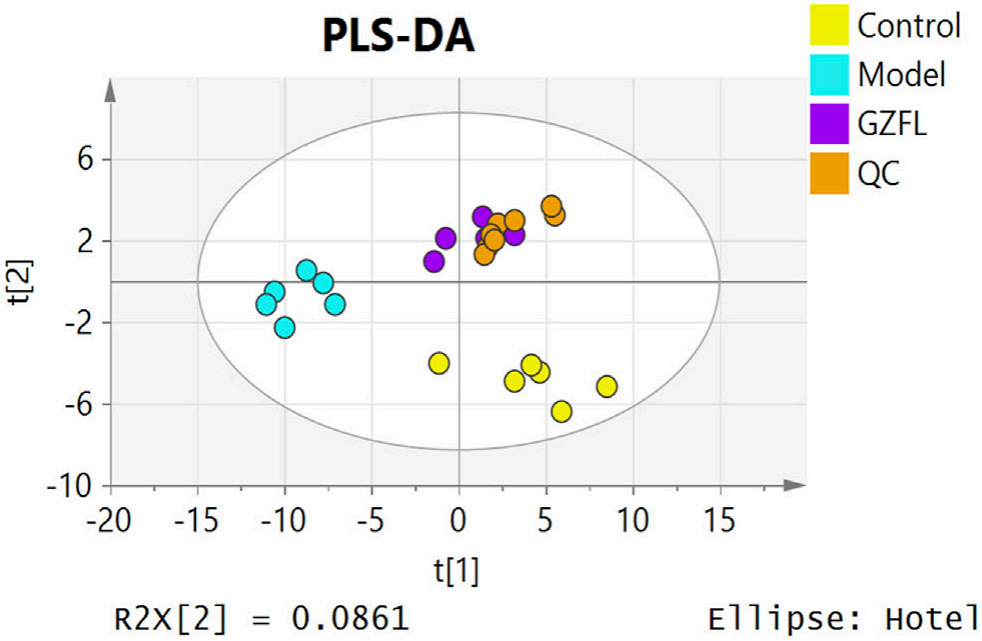
PLS-DA score plot of uterine tissue samples from three test groups (Control, Model, GZFL) and quality control (QC).

**FIGURE 6:**
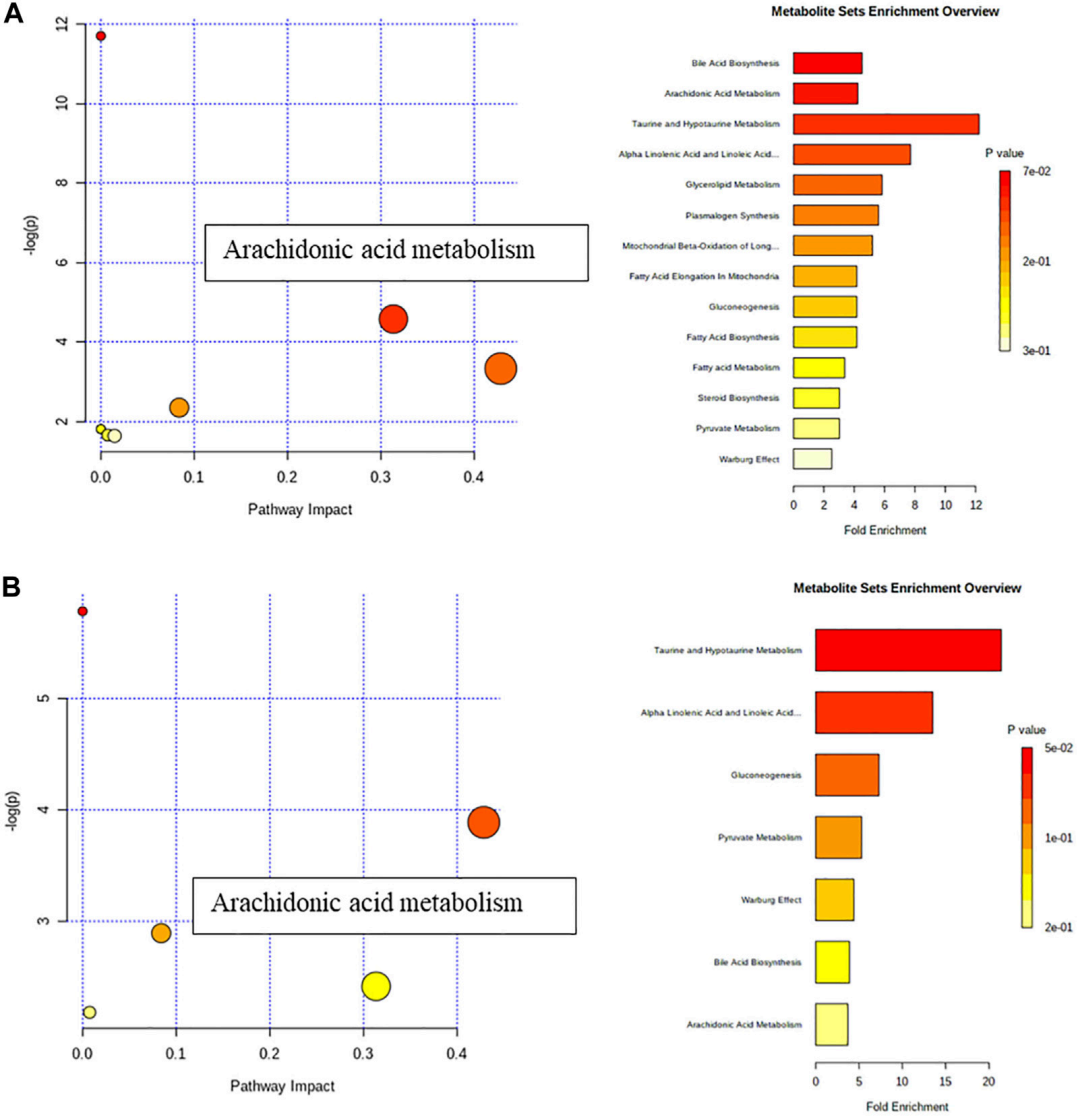
Metabolic pathway analysis **(A)** Metabolic pathway analysis between Control and Model group **(B)** Metabolic pathway analysis between Model and GZFL group.

**FIGURE 7:**
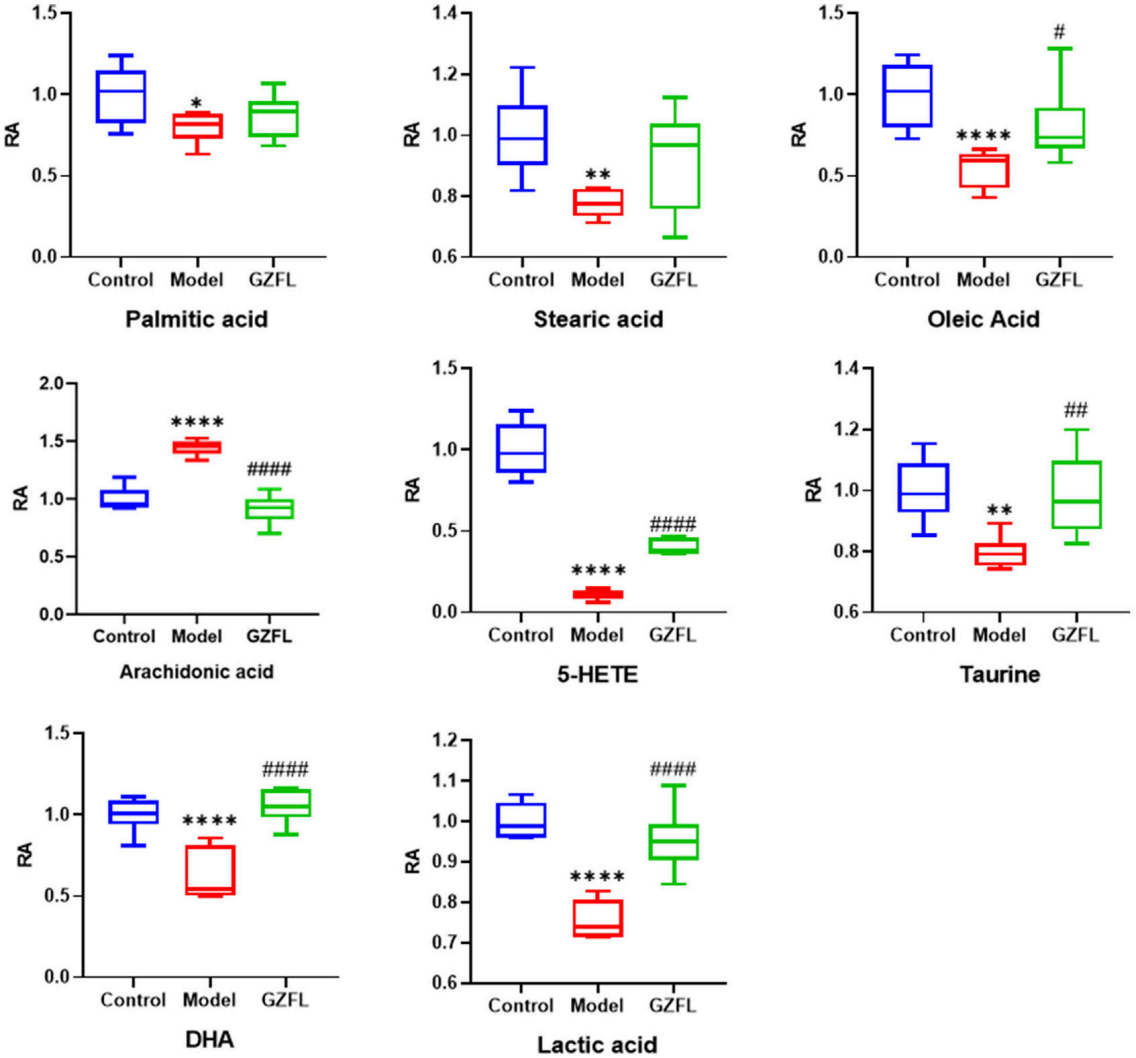
Relative abundance (RA) of the differential metabolites ( $\bar{\text{x}}$ ±*s*, n = 6, compared with blank control group: ^*^ *p* < 0.05 ^**^ *p* < 0.01 ^***^ *p* < 0.001 ^** **^ *p* < 0.0001; compared with the model group: ^#^ *p* < 0.05 ^##^ *p* < 0.01 ^###^ *p* < 0.001 ^####^ *p* < 0.0001).

**FIGURE 8:**
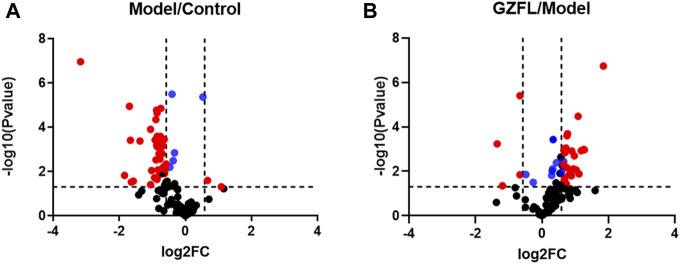
Volcano plots of compounds detected in uterine tissue for metabolomics analysis (A) Volcano plot for Model group versus Control group (B) Volcano plot for Guizhi Fuling Capsules administration group (GZFL) versus Model group. Take |FC| = 1.5 and *p*-value = 0.05 as the truncation standard. The black dots represent compounds with no significant difference, the red dots are compounds with a differential expression fold greater than 1.5 times, and the blue dots represent compounds with a significant difference but a fold change of less than 1.5 times.

**FIGURE 9:**
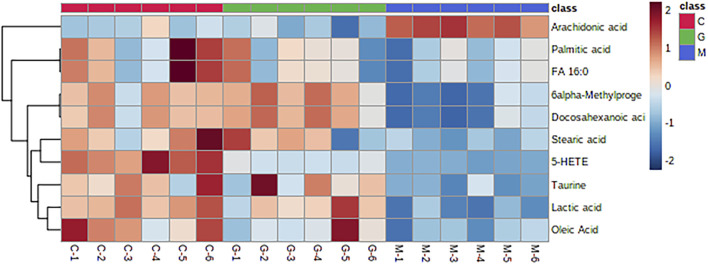
Heatmap of hierarchical clustering analysis for three test groups of Control rats (group C), Model rats (group M) and Guizhi Fuling Capsules administration rats (group G), the abscissa represents different experimental groups, the ordinate represents the differential metabolites investigated, the color represents the relative content of the corresponding metabolite in the corresponding sample, red represents the higher content in the sample, and blue represents the lower content.

Analyze the uterine sample data by PLS-DA. By observing its distribution in the mathematical model space, it was found that the PLS-DA (Figure 5) score charts have better inter-group differences and intra-group aggregation. The uterine samples of the control group, model group and Guizhi Fuling group are distinguished significantly, and there is a big difference in metabolism of each group. The results of the substitution test showed that the model has good reliability (Supplementary Figure S1). Firstly, the difference compounds are initially screened out through the condition of VIP>1, and then the ones that meet the condition of *p* < 0.05 are screened out through the *t* test. The differential compounds were analyzed on MetaboAnalyst website. The results of metabolic pathways are shown in Figure 6. The metabolic pathways mainly focus on fatty acid production, arachidonic acid and taurine metabolism. Figure 7 is a comparison diagram of the content of compounds detected in the uterine tissue of the dysmenorrhea model group and the normal control group, the Guizhi Fuling Capsule administration group and the dysmenorrhea model group. It can be found that most of the differential compounds are significantly down-regulated in primary dysmenorrhea, but after Guizhi Fuling Capsule interferes with dysmenorrhea, most of the differential compounds are significantly up-regulated. Similarly, the heat map (Figure 8) also clearly show the obvious metabolic changes between the three investigated groups. Combined with Figure 9, it can be seen that the differential compounds in uterine tissue are mainly concentrated on some saturated and unsaturated fatty acids, and this change could be reversed after drug intervention.

### Regulation in COX-2 Expression

**FIGURE 10:**
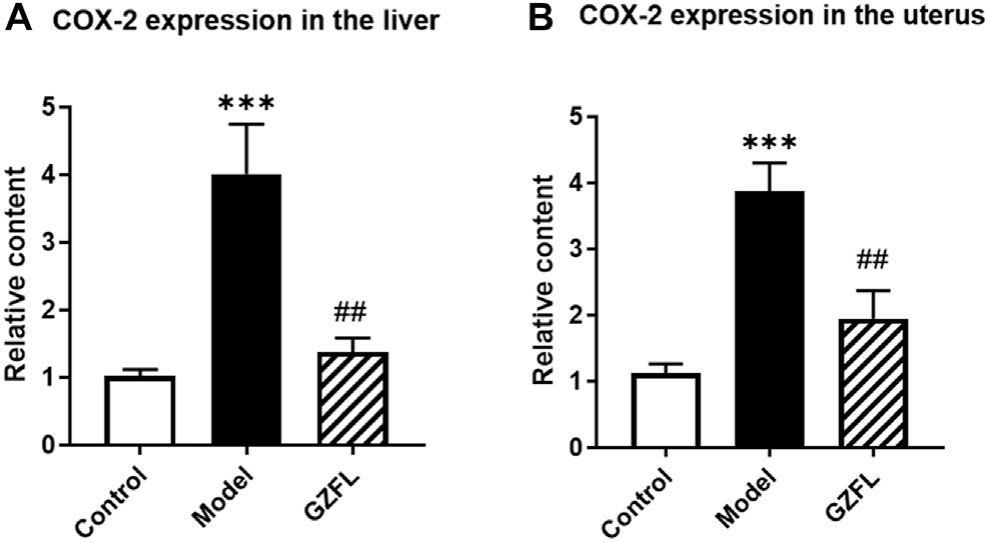
COX-2 gene expression changes in rats **(A)** COX-2 expression in liver **(B)** COX-2 expression in uterine tissue ( $\bar{\text{x}}$ ±*s*, n = 6, compared with Control group: ^***^ *p* < 0.001; compared with Model group: ^##^ *p* < 0.01).

From metabolomics analysis results, Guizhi Fuling Capsule may exert anti-dysmenorrhea effects by affecting the metabolism of fatty acids *in vivo*, such as oleic acid, stearic acid, palmitic acid, and arachidonic acid. Among them, cyclooxygenase-2(COX-2) is a rate-limiting enzyme involved in the cyclooxygenase metabolism pathway of arachidonic acid, which can catalyze the conversion of arachidonic acid to prostaglandins (PGs) and induce inflammation. Our results have found that the expression of COX-2 both in liver and uterus happened to increase significantly (about a 4-fold increase) after dysmenorrhea modeling. However, after the intervention of Guizhi Fuling Capsules, its expression was significantly reduced (about 2 times lower) (Figure 10). Therefore, we speculate that Guizhi Fuling Capsule may inhibit the conversion of arachidonic acid to *p*Gs by down-regulating the expression of COX-2, thereby inhibiting the occurrence and development of inflammation. .

## Discussion

We choose female rats to establish primary dysmenorrhea model, because female rats have a short estrus cycle, leading them suitable to study the changes in the reproductive cycle (Marcondes et al., 2002). Then, dysmenorrhea itself is a physiological phenomenon that mostly occurs within a certain age group of human beings, especially primary dysmenorrhea, which is caused by non-pelvic organic diseases. Primary dysmenorrhea is mostly caused by mental factors, and most of them occur in menarche. The average age at which Eastern women experience menarche is 12–16 years old. The three-month-old rat is equivalent to a human being 12–14 years old. Therefore, we finally chose three-month-old female rats as our model animals.

The model of experimental dysmenorrhea could be established by using estradiol benzoate and oxytocin, in order to synchronize the uterine cycle and cause artificial estrus, animals were given estrogen for 10 consecutive days to increase uterine sensitivity. After artificial estrus, oxytocin was injected to the animals to induce contraction of uterine smooth muscle and cause pain, which was manifested as writhing response. Writhing response is the most direct expression of dysmenorrhea in rats, so it is the most widely used behavioral index for the overall evaluation of visceral pain response. Studies have shown that the occurrence of dysmenorrhea is mainly related to the abnormal synthesis and release of prostaglandins in the endometrium of the menstrual period. The increased ratio of PGF2α/PGE2 leads to enhanced spastic contraction of uterine smooth muscle and dysmenorrhea (Yoshino and Ellis, 1987). PGE2 is one of the most important endogenous substances for inhibiting the inflammatory response. It has the effects of dilating blood vessels, increasing blood flow to organs throughout the body, reducing peripheral resistance of blood vessels, immunosuppression and anti-inflammatory. In the pharmacodynamics experiment, PGF2α/PGE2 in the serum of the model group rats was significantly increased, and significantly decreased after administration. β-EP has a powerful endogenous analgesic effect. It is affected by sex hormones, and participates in the regulation of reproductive endocrine. In the rat pharmacodynamics experiment, β-EP and PGE2 showed the same trend. Compared with the control group, the concentration of β-EP in the serum of the model group was significantly reduced; compared with the model group, that of the Guizhi Fuling Capsules group was significantly higher. This is the same as the results of previous studies (Sun et al., 2014).

The uterine tissue samples of dysmenorrhea rats were analyzed by metabolomics. It was found that many fatty acids had significant changes in the dysmenorrhea model, but Guizhi Fuling Capsules can reverse this change to varying degrees after intervention. Among them, arachidonic acid metabolic pathway is in a pivotal position. Arachidonic acid has many metabolic pathways, one of which is to generate prostaglandin and thromboxane through cyclooxygenase metabolism (Wang et al., 2014). Prostaglandin endoperoxidase (PTGS), also known as cyclooxygenase (COX), is the key enzyme that regulates the release of prostaglandins, including two isoforms: structural cyclooxygenase-1 (COX-1) and inducible cyclooxygenase-2 (COX-2), in which COX-2 is an inducible form only existing in inflammation. After being stimulated by inflammatory mediators, endotoxin, hypoxia factor, epidermal growth factor (EGF), COX-2 gene is induced to be highly expressed, resulting in the increase of PGI2, PGE1, PGE2 content, which could participate in inflammatory reaction. This is considered as the main source of inflammatory prostaglandins (Mitchell and Kirkby, 2019). In dysmenorrhea model rats, the expression of COX-2 in liver and uterus of rats increased significantly, and the expression of mRNA in rats from Guizhi Fuling group decreased significantly compared with the model group (Figure 10). The results suggested that Guizhi Fuling Capsules could reduce the production of prostaglandin by regulating the expression of COX-2.

Moreover, docosahexaenoic acid (DHA) is a type of n-3 unsaturated fatty acid. Recent studies have shown that when metabolized by cyclooxygenase and lipoxygenase, DHA is transformed into powerful anti-inflammatory molecules, and DHA may produce anti-inflammatory effects by inhibiting the arachidonic acid cascade (Tokuyama and Nakamoto, 2011). Lactic acid is closely related to the metabolism and polarization of macrophages. Lactic acid is a regulator of macrophage metabolism and can prevent excessive inflammation. Studies have revealed a new connection between lactic acid and the control of uterine inflammation, and found that high levels of lactic acid act on uterine GPR81 and down-regulate pro-inflammatory genes (Zhou et al., 2021). Taurine is an endogenous anti-injury substance in humans. Studies have shown that taurine has a protective effect on nonylphenol-induced uterine pathological damage in mice (Ding et al., 2018).

In summary, we speculate that Guizhi Fuling Capsules may partially reduce the synthesis of arachidonic acid through the arachidonic acid metabolic pathway, and regulate the expression of COX-2 to reduce the production of downstream prostaglandins, thereby reducing the production of inflammatory mediators release to achieve the purpose of alleviating dysmenorrhea.

## Conclusion

In a word, we have proved that Guizhi Fuling Capsules has a good effect on cold coagulation dysmenorrhea model rats, can significantly reduce the number of writhing, regulate the level of serum PGE2, PGF2α, β-EP, improve endometrial hyperplasia and ovarian function. The uterine tissue samples of dysmenorrhea rats were analyzed by metabolomics. It was found that Guizhi Fuling Capsules could reduce the interference of estradiol benzoate and oxytocin on the metabolism products of rats. Guizhi Fuling Capsules mainly played an anti-dysmenorrhea role by regulating the metabolism of arachidonic acid. More results suggest that Guizhi Fuling Capsules may inhibit the conversion of arachidonic acid into prostaglandins by down-regulating the expression of COX-2. Further experiments are needed to study the effect of Guizhi Fuling Capsules on other substances in arachidonic acid metabolism pathway.

## Data Availability

The original contributions presented in the study are included in the article/Supplementary Material, further inquiries can be directed to the corresponding authors.
