# Supplementary figures and images for: Metabolomics Study of Guizhi Fuling Capsules in Rats With Cold Coagulation Dysmenorrhea
Source: Front Pharmacol. 2021 Oct 28;12:764904. doi: 10.3389/fphar.2021.764904 (PMC8581447; doi:10.3389/fphar.2021.764904)

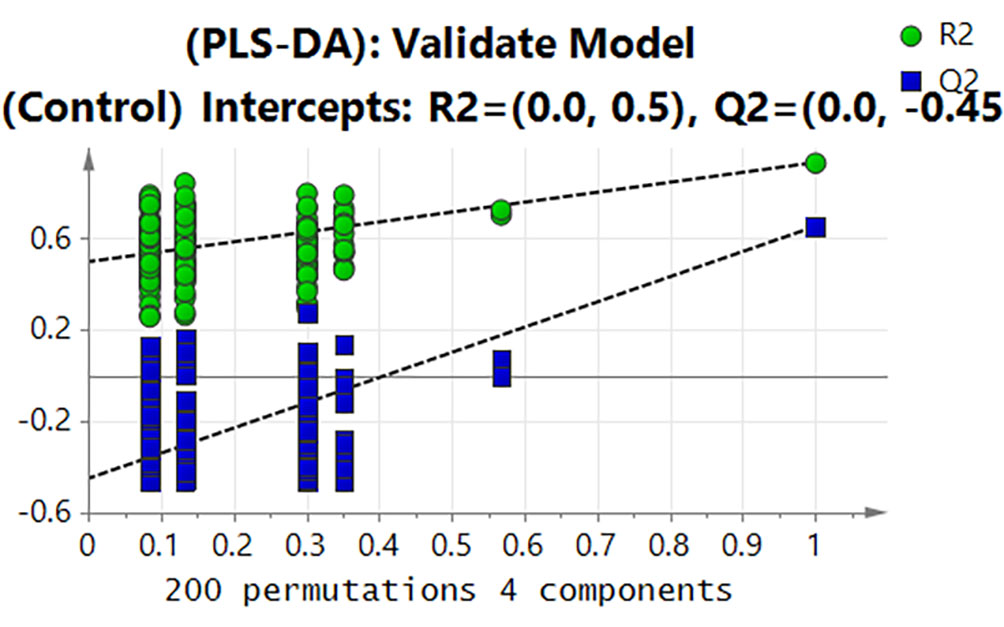

Supplement: Supplementary file 1 [file Image1.TIF]
